# The Interactions of Aquaporins and Mineral Nutrients in Higher Plants

**DOI:** 10.3390/ijms17081229

**Published:** 2016-07-29

**Authors:** Min Wang, Lei Ding, Limin Gao, Yingrui Li, Qirong Shen, Shiwei Guo

**Affiliations:** 1 Jiangsu Key Lab for Organic Waste Utilization, Nanjing Agricultural University, Nanjing 210095, China; minwang@njau.edu.cn (M.W.); 2013203033@njau.edu.cn (L.G.); 2014103113@njau.edu.cn (Y.L.); shenqirong@njau.edu.cn (Q.S.); 2 Institut des Sciences de la Vie, Université Catholique de Louvain, Louvain-la-Neuve B-1348, Belgium; lei.ding@uclouvain.be

**Keywords:** aquaporin, water transport, membrane protein, mineral nutrient

## Abstract

Aquaporins, major intrinsic proteins (MIPs) present in the plasma and intracellular membranes, facilitate the transport of small neutral molecules across cell membranes in higher plants. Recently, progress has been made in understanding the mechanisms of aquaporin subcellular localization, transport selectivity, and gating properties. Although the role of aquaporins in maintaining the plant water status has been addressed, the interactions between plant aquaporins and mineral nutrients remain largely unknown. This review highlights the roles of various aquaporin orthologues in mineral nutrient uptake and transport, as well as the regulatory effects of mineral nutrients on aquaporin expression and activity, and an integrated link between aquaporins and mineral nutrient metabolism was identified.

## 1. Introduction

Aquaporins, small integral proteins that belong to the ancient family of major intrinsic proteins (MIPs), have been found in all kingdoms of life. In plants, aquaporins reside in the plasma membrane and tonoplast and play important roles in plant water relations by facilitating the transport of water across biological membranes and regulating osmotic potential and hydraulic conductivity [1,2]. The regulatory roles of aquaporins in cellular water transport have been reported in previous studies [3,4,5,6]. In general, the molecular mechanisms of water transport across plasma membranes regulated by aquaporins are mainly attributed to co-translational and post-translational modification, aquaporin gating, and tetramer assembly and cellular trafficking of plasma membrane intrinsic proteins [1,4].

Based on amino acid sequence similarities, aquaporins are classified into seven subfamilies. The plasma membrane intrinsic proteins (PIPs) and the tonoplast intrinsic proteins (TIPs) are the most abundant aquaporins in the plasma membrane and tonoplast, respectively [3,4]. The nodulin 26-like intrinsic proteins (NIPs), are located in the peribacteroid membrane of nitrogen-fixing symbiotic root nodules of leguminous plants and are also present in the plasma membrane of other species [7]. The small basic intrinsic proteins (SIPs) are small proteins mainly localized in the ER membrane [8], and the uncharacterized X intrinsic proteins (XIPs) are plasma membrane aquaporins that function in the transport of uncharged substrates [9]. The hybrid intrinsic proteins (HIPs) and the glycerol facilitator (GlpF)-like intrinsic proteins (GIPs) are present exclusively in moss [10]. The large number of plant aquaporins has been explained by their importance in regulating plant metabolic processes under various physiological states and environmental conditions [11]. For example, aquaporins are essential for plant defence responses against biotic and abiotic stresses, such as drought [12], salt stress [13,14], cold [15,16], nutrient deprivation [17], heavy metals [18,19], and pathogen infection [20,21]. Aquaporins play complex integrated roles in the response to different environmental stressors and are involved in plant growth and metabolic processes. PIPs and TIPs are involved in drought, salt, and cold stress through hydraulic conductivity and transpiration regulation, while TIPs and NIPs are involved in biotic stress, predominantly nutrient homeostasis between pathogens and host plants [22].

Mineral nutrients, which are usually present in the soil solution in organic and inorganic forms, are essential for plant growth and production. Physiological analysis indicated that ion uptake was regulated by transporters in the root plasma membranes, and there is a strong interaction between mineral nutrients and water status in which mineral nutrient uptake is accompanied by water absorption. In addition to facilitating water diffusion, a number of aquaporins have also been shown to transport other small neutral molecules, such as urea [23,24], ammonia (NH_3_) [25,26], carbon dioxide (CO_2_) [27,28,29], boric acid [17,30,31], silicic acid [32,33,34], lactic acid [35], hydrogen peroxide (H_2_O_2_) [9,36,37,38], and other molecules with physiological significance [39]. Aquaporin trafficking and their subcellular relocalization act as a critical point for regulating the internal redistribution of mineral nutrients by transporting them from the endoplasmic reticulum (ER) to the plasma membrane via the Golgi apparatus, as well as undergoing repeated cycles of endocytosis and recycling through the early endosome to the multivesicular body/prevacuolar compartments before eventually being targeted to the vacuole [4]. However, the molecular and cellular mechanisms underlying the interactions of aquaporin and mineral nutrients should be further investigated. In this review, the role of aquaporins in maintaining the plant water and mineral nutrient status is discussed, and the cellular aspects of plant aquaporin functions and regulation of mineral nutrients are also extensively reviewed.

## 2. Nitrogen (N)

Nitrogen (N), one of the most important mineral nutrients in higher plants, is involved in plant metabolism as a constituent of amino acids, proteins, nucleic acids, lipids, chlorophyll, co-enzymes, phytohormones, and secondary metabolites [40,41]. The interaction between aquaporins and N assimilation was first identified following the observation that the expression of several aquaporin genes responded to different N sources, such as *AtTIP2;1*, which was up-regulated by N starvation or NH_4_^+^ supply [26], while both *PtdPIP1;2* and *PtdSIP1;2* were down-regulated under high N fertilization levels [42]. Aquaporins have been suggested to be involved in water transport in response to nitrogen availability [42,43,44]. In our previous study, a high N (mixture of NH_4_^+^ and NO_3_^−^) supply enhanced aquaporin (AQP) expression and decreased root aerenchyma and lignin, resulting in a high water absorption rate [44], which was consistent with results that show that high N supply increases root hydraulic conductivity and AQP expression in rice plants [43]. The possible mechanisms of aquaporin regulated hydraulic conductivity in response to N availability may be attributed to the changes in aquaporin abundance and activity [43]. Aquaporins play an important role in N absorption, mobilization, and detoxification, as well as other nitrogen metabolic processes in higher plants [23]. The PIP, NIP, and TIP subfamilies have been shown to transport N compounds, including ammonia and urea [23,24]. 

### 2.1. Nitrate

Nitrate is the major inorganic N source absorbed by upland plants, and the process of nitrate uptake and metabolism is tightly associated with water utilization, which is regulated by AQP. Nitrate was suggested to be a critical signalling factor for radial water fluxes in the roots [45,46,47], and the increased root hydraulic conductivity (Lpr) by nitrate was shown to correlate with up-regulation of aquaporin expression [46,47,48]. In maize, the expression of *ZmPIP1;5b* was strongly up-regulated by nitrate [49], and in tomato plants, several AQP genes were up-regulated by the nitrate supply [50], which can mediate and control the increased water influx into the root cells.

Recently, Li et al. [51] showed that Lpr and PIP expression were controlled by both exogenous and internal nitrate concentrations in *Arabidopsis*, and Lpr and PIP expressions were higher under 5 mM NO_3_^−^ than 0.5 mM NO_3_^−^. In *nrt2.1* (the high-affinity NO_3_^−^ transporter) mutant plants, NO_3_^−^ content decreased in both the roots and shoots, which resulted in decreases in Lpr and PIP expression, indicating that the nitrate supply was positively correlated with enhanced root AQP activity and Lpr. However, the interactions between nitrate and aquaporins vary over time. In the short term, hours to days, nitrate induced aquaporin expression [50], while over multiple days, root morphology and proliferation were significantly altered by the nitrate supply, resulting in increases in root nitrate acquisition [52,53].

### 2.2. Ammonia/Ammonium

Root NH_4_^+^ uptake occurs mainly via ammonium transporters (AMT) in the plasma membrane, while NH_3_ has been proposed to enter the cells by free diffusion in higher plants. Transport of NH_3_/NH_4_^+^ and urea into the vacuole would allow for N storage and eliminate their toxicity to the plant [54], and when N was needed, the stored nitrogen could be remobilized by a passive, low-affinity transport pathway, which may involve the TIP proteins [23]. Indeed, several tonoplast intrinsic proteins (TIPs) have been shown to facilitate the NH_3_ transport, such as ZmTIP1;1 and ZmTIP1;2 [55]. TIPs from wheat (TaTIP2;1) and *Arabidopsis thaliana* (AtTIP2;1 and AtTIP2;3) not only function as water-conducting membrane pores but also facilitate the transport of NH_3_ across membranes and therefore mediate the remarkable loading and acid-trapping of the protonated form (NH_4_^+^) in the vacuole [26,56]. However, the importance of the channel pores in ammonia transport by TIP2;2 from wheat has been challenged by the finding that NH_3_ is not transported with water but through a separate pathway [25]. The crystal structure of an NH_3_ permeable aquaporin AtTIP2;1 demonstrated that an intriguing water-filled side pore, next to the substrate-binding histidine, is involved in deprotonating ammonium ions, thereby increasing the permeation of NH_3_ [57].

**Figure 1:**
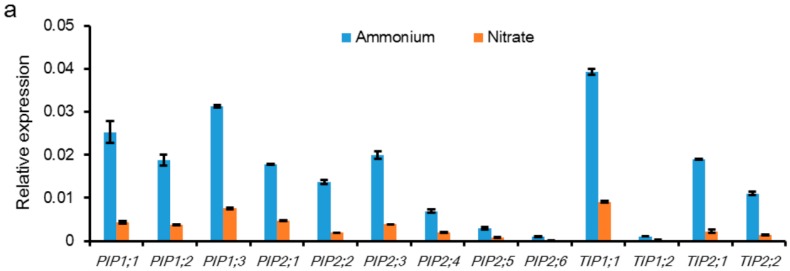

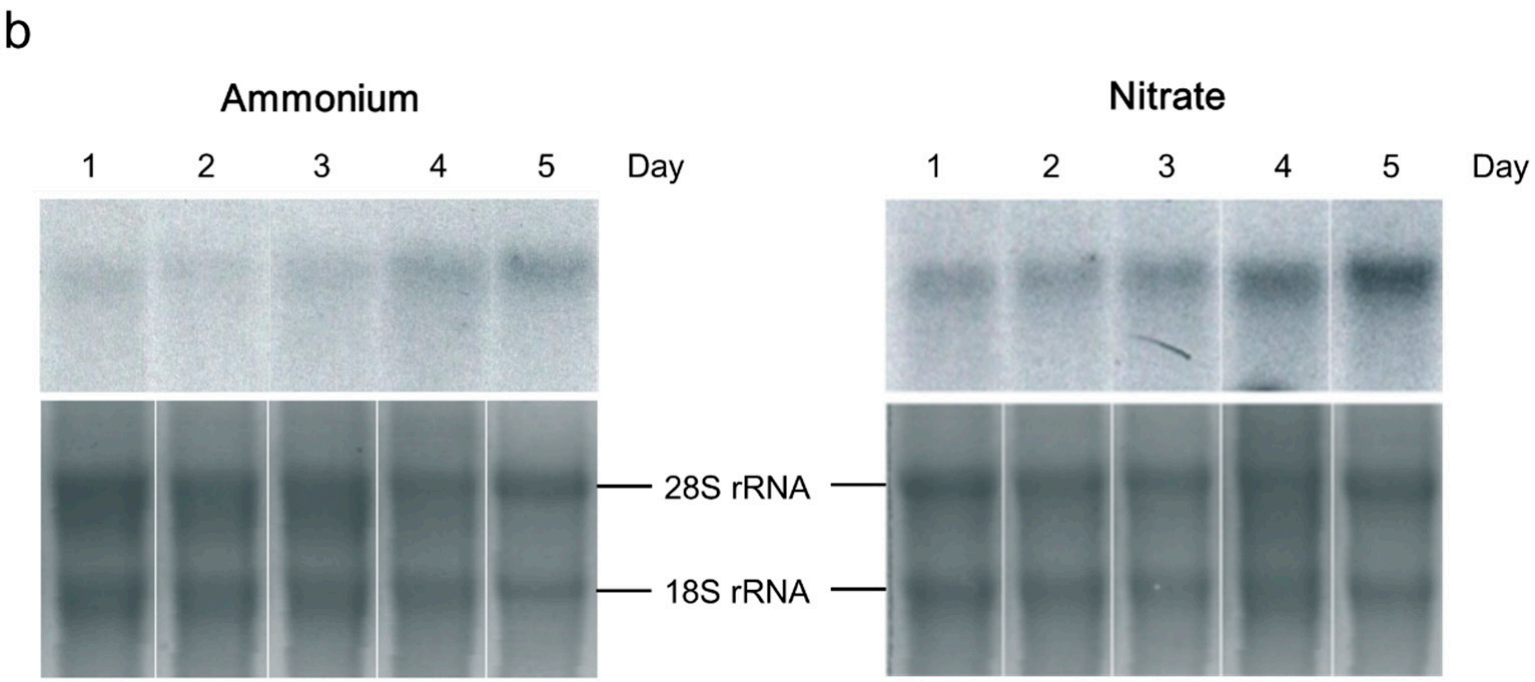
Effect of different nitrogen sources (ammonium vs. nitrate) on aquaporin (AQP) expression in rice (**a**) and French bean plants (**b**). Rice plants were supplied with 2.5 mM ammonium [(NH_4_)_2_SO_4_] or nitrate [Ca(NO_3_)_2_] for two weeks. Root samples were collected for RNA isolation, and quantitative real-time PCR (q-RT-PCR) was performed to detect the relative expression of the plasma membrane intrinsic proteins (PIPs) and tonoplast intrinsic proteins (TIPs) [60]; (**b**) French bean plants were grown in a split-root system, in which half of the roots were supplied with 5 mM ammonium [(NH_4_)_2_SO_4_] or nitrate [Ca(NO_3_)_2_]. The PIP1 aquaporin expression in the roots was determined via Northern blot analysis until day 5 after the treatments [59].

Additionally, there was a potential correlation between ammonium uptake and water absorption, which was regulated by AQP. In rice plants, ammonium could increase the expression of *PIP* and *TIP* genes in the roots and resulted in a higher water uptake rate compared with that of nitrate (Figure 1a). Under water stress, ammonium increased drought tolerance of rice plants by inducing aquaporin expression and/or activity, which corresponded with increased root water uptake ability [58]. However, in French bean plants with a ‘one shoot-two roots’ split root system, Guo et al. [59] demonstrated that the mRNA expression of PIP1 was higher in the roots supplied with nitrate than those supplied with ammonium (Figure 1b). Generally, rice prefers ammonium nutrition while beans prefer nitrate nutrition, demonstrating that AQP expression is upregulated under favoured nitrogen nutrition. These results suggested that ammonium and nitrate differentially regulated water uptake and AQP in different plant species.

### 2.3. Urea

Urea is a major N fertilizer used in agricultural production and is also a naturally occurring and readily available N source in soil. Urea is an uncharged small solute and passes through plant membranes via AQP [49,61,62,63], and members of the PIP, NIP, and TIP subfamilies have been shown to facilitate urea crossing membranes [23,24,64]. NIPs and PIPs, localized to the plasma membrane, function in urea movement between the apoplast and the symplast of plant cells [33,65,66]. In comparison, TIPs were targeted mainly to the tonoplast or other endo-membranes and are involved in equilibrating urea concentrations between different cellular compartments [23].

In *Arabidopsis*, several native NIPs, such as AtNIP6;1 and AtNIP5;1, were shown to transport urea, and AtNIP6;1 was also predicted to conduct substantial amounts of ammonia [64], and AtNIP5;1 was identified to transport boron acid [67]. However, AtNIP5;1 was identified to facilitate urea uptake only under B deficiency, in both high and low urea concentrations [67]. In maize plants, ZmNIP2;1, ZmNIP2;4, and ZmTIP4;4 were found to be involved in urea transport and played critical roles in urea uptake and movement, and stabilized urea concentrations in the tonoplast [23,63]. CsNIP2;1, a plasma membrane transporter from *Cucumis sativus*, facilitates urea uptake and internal transport during N remobilization and N delivery in plants [62]. In maize roots, the ZmPIP1;5, an aquaporin transport for water and urea, diverges from other PIP membranes by urea transport activity [49], and the expression of *ZmPIP1;5* is induced by nitrate and modulated during the day-night cycle. Vacuoles could be used for short-term urea storage to avoid toxicity in the cytoplasm; this process was regulated by TIPs, which contribute to urea remobilization from the vacuole and equilibration within the cell [24,68]. Under nitrogen deficient conditions, expression of *ZmNIP2;1* and *ZmNIP2;4* was not affected, whereas the expression of *ZmTIP4;4* increased significantly in the roots and expanded leaves, suggesting that ZmTIP4;4-regulated urea transport was essential for unloading vacuolar urea across the tonoplast under N starvation conditions [63]. Moreover, AtTIP1;1, AtTIP1;2, AtTIP2;1, and AtTIP4;1, which are different from the high-affinity H^+^/urea symporter AtDUR3, provide a less concentration- and pH-dependent pathway for urea transport from the external growth medium into the cytosol or from the cytosol into the vacuole [23,61]. AtTIP1;3 and AtTIP5;1, the only highly expressed pollen-specific aquaporins, function in N remobilization via transport of mitochondrial urea to the cytoplasm [61,69].

Aquaporins are tightly linked with N metabolism in higher plants, and the PIP, NIP, and TIP subfamilies have been shown to transport NH_3_ and urea, and maintain the balance between the cytoplasm and vacuole. Understanding the principles of N compounds passing through the plasma membrane by aquaporins allow us to modulate the N uptake and utilization, and improve the nitrogen use efficiency in plants. Nitrate and ammonium were different in regulation of plant water uptake and AQP expression depending on the plant species. Nitrate is suggested to be a critical signalling factor to induce PIPs expression and increase root hydraulic conductivity in nitrate preferred plants, while ammonia increases PIPs and TIPs expression and water uptake in ammonia preferred plants. The regulation of AQP by different nitrogen forms provides an effective pathway to increased plant water stress and water use efficiency in plants.

## 3. Phosphorus (P)

Phosphorus (P) is necessary for the synthesis of nucleic acids, which contains the genetic code of the plant for production of proteins and other compounds essential for plant structure, seed yield, and genetic transfer. A number of studies have indicated that the enhancement in plant growth with P fertilization is associated with an increased capacity of the plants to transport water [70,71]. The activity or density of aquaporins in the plasma membrane of root cells is diminished during nutrient stress, such as N- and P deprivation [48].

P is involved in root water uptake by altering aquaporin expression and/or activity [48,72]. P deficiency reduced aquaporin activity or abundance in the root plasma membrane [48] and was associated with a decrease in water uptake [72], which was attributed to phosphorylation of the plant aquaporins [73]. In *Arabidopsis* roots, the changes in the phosphorylation status of PIP aquaporins were positively correlated to changes in root hydraulic conductivity under NaCl, NO, and N and P starvation treatments [74]. Additionally, plants often exhibit disruption of water transport that is associated with enhanced ethylene production, which modulates root hydraulic conductivity by affecting the aquaporin activity under P deficient conditions [75,76]. As plant aquaporins are regulated by cytosolic pH and free Ca^2+^ activity [77], ethylene can elicit a rapid increase in cytosolic Ca^2+^ concentration by activating the Ca^2+^-permeable channels [78], as a result of inhibiting aquaporin activity. In sorghum plants, the root hydraulic conductivity of water-stressed plants with a sufficient P supply recovered faster than that of plants without a P supply, suggesting that sufficient P could increase AQP expression and/or activity after water recovery [79]. Arbuscular mycorrhizal (AM) fungi, which formed symbiotic associations with host plants, can uptake and deliver inorganic P to the host through hyphal networks. Under water stress, AM symbiosis can increase the tolerance of plants by regulating the AQP gene expression, osmotic adjustments, and plant growth [80,81].

The role of P on aquaporins is mainly focused on its phosphorylation functions by regulating aquaporin activity and abundance, and corresponds with regulated root hydraulic conductivity and water uptake. Further studies are needed to elucidate the specific functions of AQP genes regulated by AM symbiosis, in order to reveal the exact mechanism of AM symbiosis to deliver P and alter plant adaptation to environmental stressors.

## 4. Potassium (K)

Potassium ion (K^+^), the most abundant cation in higher plants, functions in osmo-regulation, cation-anion balance, stomatal movement, photosynthesis, energy transfer, carbohydrate phloem transport, enzyme activation, and protein synthesis, as well as stress resistance [41]. As K^+^ is the major osmolyte, its uptake will be accompanied by water flux through the aquaporins, and there was a positive correlation between K absorption and water uptake [82]. It was suggested that aquaporins could function as turgor sensors to modulate the conductance of K^+^ channels [83]. Transcripts encoding aquaporins were strongly affected by K^+^ starvation, even without water stress [84]. In *Arabidopsis*, iterative group analysis (iGA) identified 12 aquaporin genes in the shoots and 15 genes in the roots that were significantly up-regulated after K^+^ resupply [85]. The trafficking and activity of plasma membrane aquaporin PIPs is regulated by the SNARE SYP121, a plasma membrane resident syntaxin involved in vesicle trafficking, signaling, and regulation of K^+^ channels [86,87]. SYP121 plays a role in the regulation and maintenance of membrane osmotic water permeability through a coordinated regulation of the plasma membrane density of both PIP and K^+^ channels in membrane delivery and recycling [86].

The aquaporins may participate in ion homeostasis at the whole plant level by regulating the ratio of apoplastic/symplastic water flow and thus directing solute flux through different plant tissues. In onion roots, water transport was sensitive to inhibitors of aquaporins and K^+^ channels, and the decrease in hydraulic conductivity after K^+^ channel inhibitor treatment indicates that K^+^ fluxes are involved in aquaporin activity in the plasma membrane [88]. In *Arabidopsis* roots, the expression of genes encoding water channels of the aquaporins PIP1b, PIP2b, and TIP, as well as the K^+^ transporter HAK5 were reduced after K^+^ channel inhibitor (CsCl) treatment [89], suggesting that K^+^ channel blockers could reduce the hydraulic conductivity of the plasma membrane by down-regulating or blocking water channels.

It has been reported that aquaporins and K^+^ channels can function as plant osmo-regulators to maintain cytosolic osmolarity and increase the tolerance of the plant to drought or other stressors [90,91]. In rice, the expression of PIP and K^+^ channels responded similarly to K deficiency and water stress, in which expression of PIPs and K^+^ channel-encoding genes was induced by K^+^ starvation and down-regulated by water deficit during a short time, suggesting that aquaporins and K^+^ channels are functionally co-regulated during cell turgor regulation [90]. Loading K^+^ into the plant xylem could regulate the xylem hydraulic conductivity, which can help maintain cell turgor, stomatal aperture, and gas exchange rates, as a result of increasing drought tolerance [92,93]. During drought stress, plants modulate their water and ion uptake capacities by regulating aquaporins and K^+^ channels at the transcriptional level to respond to the water deficiency [90,94,95].

Aquaporins participate in whole plant ion homeostasis and act as turgor sensors to modulate the K^+^ channels. Aquaporins and K^+^ channels can function as plant osmo-regulators to maintain cytosolic osmolarity and increase tolerance to drought stress, which corresponds with rapid recovery of the shoot water status, cell turgidity, and thus plant growth. The coordination of aquaporins and K^+^ transport in plants during different stressors and physiological states, may be regulated by different signalling pathways.

## 5. Calcium (Ca)

Calcium (Ca) is an essential macronutrient that functions in the cell wall and membranes, acting as a counter-cation for inorganic and organic anions in the vacuole, as well as a secondary messenger in cell signal transduction [41,96,97]. Generally, Ca^2+^ enters the root apoplast via the mass flow from the soil solution [41], suggesting that transpiration-regulated water flow may be involved in Ca^2+^ delivery and storage, which could be regulated by aquaporins [97]. Conversely, Ca^2+^ could affect AQP activity and/or expression, and aquaporin expression was suppressed by Ca^2+^ starvation [84]. It has been reported that the inhibition of maize root water transport by HgCl_2_ was detected only in the presence of Ca^2+^ in the nutrient solution, suggesting that Ca^2+^ is involved in regulating aquaporin activity [98].

Several studies demonstrated that the aquaporin functions could be regulated by Ca^2+^ [99,100] and triggered by environmental stressors [101]. Under water stress, the expression or activity of aquaporins was affected [94,98], and this process could be facilitated by excess Ca^2+^ [102]. Salt stress decreased water transport through the plasma membrane and the root cortical cells by reducing Hg-sensitive aquaporin activity, and the ameliorative effect of Ca^2+^ on salt stress was related to aquaporin function [72,103,104]. In pepper plants, cytosolic Ca^2+^ decreased after long-term exposure to salt stress with a corresponding overall inhibition of aquaporins [105]. Reversible phosphorylation, a potential mechanism for plant aquaporin regulation during development and in the response of plants to environmental stimuli [73,106,107], could be regulated by Ca^2+^, indicating a link between aquaporin regulation and Ca^2+^ signalling [105].

In guard cells, extracellular Ca^2+^ is involved in stomatal movement by acting as an elicitor (second messenger) or aquaporin blocker, which may initiate the signal cascade and lead to the post-transcriptional regulation of aquaporins or directly block aquaporins [108]. The aquaporin gate was regulated by cytosolic Ca^2+^ transport, especially the opening and closing of verapamil-sensitive Ca^2+^ channels [104]. In vitro phosphorylation of the aquaporin PM28A was directly dependent on submicromolar Ca^2+^ concentrations [99]. Ca^2+^ is involved in plasma membrane aquaporin regulation via a chain of processes within the cell, but its effects are not due to alteration of the stability of the plasma membrane [104].

Cytosolic Ca^2+^ transport and Ca^2+^ channels might directly regulate water flow by acting on aquaporins, which would affect nutrient movement through the plant. In the response of plants to environmental stimuli, the functions of aquaporin could be regulated by Ca^2+^ via reversible phosphorylation. However, the regulation mechanism of Ca^2+^ on aquaporins and its physiological role in whole plant conditions remains to be established.

## 6. Boron (B)

Boron (B) is an essential micronutrient for plant growth and development, especially for the structure and function of the plant cell wall [41]. B deficiency and toxicity in plants results in a significant reduction in quality and yield of many crops worldwide [109,110]. Aquaporins have been shown to function in B transport in higher plants [111,112], and are required for normal plant growth under B deficiency and toxic conditions [17,113,114].

AtNIP5;1, a boric acid channel that belongs to the major intrinsic proteins (MIPs), is predominantly expressed in epidermal, cortical, and endodermal cells [17,30]. Under B deficiency, *AtNIP5;1* expression was strongly up-regulated, which is critical for efficient B transport into the roots [17]. In *nip5;1* mutants of *Arabidopsis thaliana*, both root and shoot growth were inhibited under B deficiency [17], indicating that NIP5;1 was essential for the overall B uptake that was required for plant growth and development under B limitation. *AtNIP6;1*, which is homologous to *AtNIP5;1*, was shown to facilitate the rapid penetration of boric acid across the membrane and normal distribution of boric acid in plant tissues, but it is completely impermeable to water [113]. Similarly, the water channel OsNIP3;1 was also found to be a B-inducible channel in rice involved in B uptake and distribution [115].

In barley, the tolerance to excessive soil B is controlled by downregulated expression of *HvNIP2;1* to reduce B uptake and leaf blade B accumulation. Expression of *Bot1*, a *BOR1* ortholog that provides B tolerance to barley, was induced to eliminate B from the roots and sensitive tissues [114]. HvNIP2;1 is essential for B toxicity tolerance in barley in combination with Bot1. AtTIP5;1 plays a critical role in the B transport pathway possibly via vacuolar compartmentation, and the overexpression of *AtTIP5;1* may facilitate the elimination of B toxicity in plants [116]. OsPIP1;3, OsPIP2;4, OsPIP2;6, and OsPIP2;7, members of the major intrinsic proteins (MIPs) family, were involved in both influx and efflux of B transport, and their expressions were strongly upregulated under B toxicity [117,118]. Briefly, aquaporins are essential for reducing the accumulation of toxic boric acid levels in plant tissues [9].

Aquaporins were involved in B uptake and distribution, and PIP, NIP, TIP, and XIP subfamilies have been shown to transport boric acid. Under B deficiency, NIPs are essential for efficient B uptake and distribution that is required for plant growth and development. Whereas under B toxicity, NIPs, TIPs, and PIPs are involved in reducing the accumulation of toxic boric acid levels in plant tissues. Manipulation of these aquaporins could be highly useful in improving plant tolerance to B deficiency or toxicity.

## 7. Silicon (Si)

Silicon (Si), the second most abundant element in the earth’s crust, is important for plant growth and development. Si is beneficial to the mechanical and physiological properties of plants and helps plants to overcome biotic and abiotic stress [34,119,120,121]. Under salt stress, Si can improve plant tolerance through enhancing root water uptake which contributes to the regulation of aquaporin activity and gene expression [122,123]. The Si uptake by plants in soil solution is through silicic acid [Si(OH)_4_], an uncharged molecule [34]. Silicic acid enters the plant roots mainly by water flow via the apoplastic and symplastic pathways, and the symplastic pathway involves the presence of water channels, mainly NIPs [124]. In rice, two Si transporters, Lsi1 and Lsi2, have been shown to be involved in Si uptake. Lsi1 is localized on the distal side of the plasma membrane of the exodermal and endodermal cells and functions as an influx transporter [33,125], whereas Lsi2 is located on the proximal side of the same root cells and functions as an efflux transporter [125,126]. The combination of Lsi1 and Lsi2 enables rice to efficiently transport silicic acid from the soil solution into the xylem of the roots. Lsi1 (also named OsNIP2;1) belongs to the NIP subfamily of aquaporins, and previous studies have shown that NIP proteins are permeable to a wide range of substrates, such as silicic acid [33], arsenite [125], boric acid [17], urea and formamide [64], glycerol [127], lactic acid [35], as well as selenite [128]. Lsi6 (OsNIP2;2), which is localized in the xylem parenchyma cells of leaf blades and sheaths, was also identified as responsible for Si xylem unloading [129]. In barley, a Lsi1 ortholog of HvLsi1 (HvNIP2;1), localized in the plasma membrane of epidermal cells and all cortical cells in roots, was identified as a Si influx transporter and shown to be involved in the radial transport of Si through the epidermal and cortical layers of the basal roots [32]. 

Si absorption is facilitated by NIPs, and two Si transporters have been identified that are involved in Si uptake, Lsi1 and Lsi2, which function as an influx and efflux transporter, respectively. Cooperation of Lsi1 and Lsi2 is required for the efficient transport of Si. The identification of Si transporters provides an insight into the Si uptake system in plants and a new approach for producing crops with high resistance to various biotic and abiotic stresses by genetic modification. To further elucidate the Si accumulation mechanism and understand the critical role of Si at the whole plant level, molecular and physiological characterization of Si transporters in different plant species is required in the future. 

## 8. Conclusions and Future Perspectives

**Figure 2:**
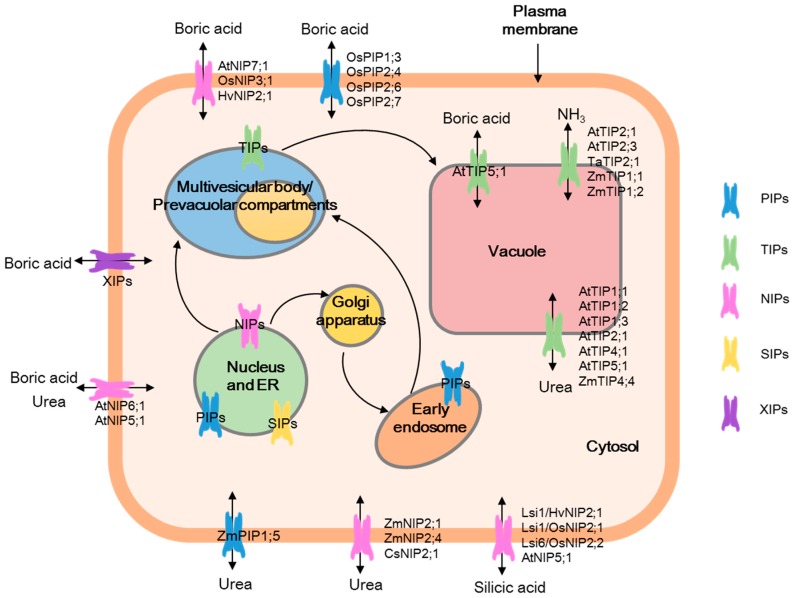
The multiple cellular functions of plant aquaporins in mineral nutrition. The figure illustrates the variety of transporter functions of aquaporins in various subcellular compartments. The different subclasses of aquaporins are identified in different colours. The plasma membrane intrinsic proteins (PIPs) might be involved in the internal re-distribution of mineral nutrients by transporting them from the endoplasmic reticulum (ER) to the plasma membrane via the Golgi apparatus. Moreover, PIPs also undergo repeated cycles of endocytosis and recycling through the early endosome to the multivesicular body/prevacuolar compartments before eventually being targeted to the vacuole. PIPs primarily facilitated urea and boric acid transport, while tonoplast intrinsic proteins (TIPs) are principally involved in urea, NH_3_, and boric acid transport, and nodulin 26-like intrinsic proteins (NIPs) are involved in urea, boric acid, and silicic acid transport.

A wide range of selectivity profiles and regulatory properties allow aquaporins to be involved in multiple functions in plant growth and development, such as water transport, and nitrogen, carbon, and micronutrient acquisition. Aquaporins, mainly PIPs, TIPs, and NIPs, have been shown to facilitate the transport of plant mineral nutrients across plasma membranes and cell organelles (Figure 2), such as ammonia, urea, boric acid, and silicic acid. Aquaporins are responsible for ensuring different mineral nutrient availability for the plant and play essential roles in mineral nutrient absorption, mobilization, detoxification, and homeostasis. There is a tight link between plant aquaporins and mineral nutrients. Aquaporin expression is regulated by mineral nutrient availability and plant species. Aquaporin function can be regulated by mineral nutrients in the response of plants to environmental stimuli, such as drought and salt stress, nutrient deficiency, and toxicity. Understanding the interactions between aquaporins and mineral nutrients allow us to modulate the water and mineral nutrient uptake and utilization, and improve the water and nutrient use efficiency in plants, as well as increase tolerance to biotic and abiotic stress. In the future, attention should be focused on:

(1) The functions of aquaporins in the transport of other novel putative substrates, such as Mg, S, and other micronutrients, which await further investigation.
(2) New aquaporin subclasses and unknown functions of aquaporins recently discovered in certain plant species should be deciphered.
(3) Investigation of the interactions between water and mineral nutrient transport, as well as interactions between different mineral nutrients regulated by aquaporins will be required.
(4) The role of aquaporins during biotic and abiotic stress, and the relevance of altered aquaporin expression for biotechnological improvement of plant tolerance must be explored.
(5) Aquaporin functions need to be further investigated concerning whole plant physiology, which requires a better understanding of how the various aquaporin transport activities are coupled with plant mineral nutrient transport proteins.
